# DNA conformational dynamics in the context-dependent non-CG CHH methylation by plant methyltransferase DRM2

**DOI:** 10.1016/j.jbc.2023.105433

**Published:** 2023-11-04

**Authors:** Jianbin Chen, Jiuwei Lu, Jie Liu, Jian Fang, Xuehua Zhong, Jikui Song

**Affiliations:** 1 Department of Biochemistry, University of California, Riverside, California, USA; 2 Department of Biology, Washington University in St. Louis, St. Louis, Missouri, USA

**Keywords:** non-CG methylation, CHH methylation, CWW motif, DNA deformation, DNA conformational dynamics, DRM2, plant DNA methylation, temperature-dependent DNA methylation, substrate specificity, flanking sequence preference

## Abstract

DNA methylation provides an important epigenetic mechanism that critically regulates gene expression, genome imprinting, and retrotransposon silencing. In plants, DNA methylation is prevalent not only in a CG dinucleotide context but also in non-CG contexts, namely CHG and CHH (H = C, T, or A) methylation. It has been established that plant non-CG DNA methylation is highly context dependent, with the +1- and +2-flanking sequences enriched with A/T nucleotides. How DNA sequence, conformation, and dynamics influence non-CG methylation remains elusive. Here, we report structural and biochemical characterizations of the intrinsic substrate preference of DOMAINS REARRANGED METHYLTRANSFERASE 2 (DRM2), a plant DNA methyltransferase responsible for establishing all cytosine methylation and maintaining CHH methylation. Among nine CHH motifs, the DRM2 methyltransferase (MTase) domain shows marked substrate preference toward CWW (W = A or T) motifs, correlating well with their relative abundance in planta. Furthermore, we report the crystal structure of DRM2 MTase in complex with a DNA duplex containing a flexible TpA base step at the +1/+2-flanking sites of the target nucleotide. Comparative structural analysis of the DRM2-DNA complexes provides a mechanism by which flanking nucleotide composition impacts DRM2-mediated DNA methylation. Furthermore, the flexibility of the TpA step gives rise to two alternative DNA conformations, resulting in different interactions with DRM2 and consequently temperature-dependent shift of the substrate preference of DRM2. Together, this study provides insights into how the interplay between the conformational dynamics of DNA and temperature as an environmental factor contributes to the context-dependent CHH methylation by DRM2.

Cytosine C-5 DNA methylation is an evolutionarily conserved epigenetic mechanism that plays an essential role in regulating gene expression and genome stability (1). Dysregulation of DNA methylation leads to developmental defects and/or diseases in animals and plants (2, 3), highlighting the importance of functional regulation for this modification. It has been well established that plants and animals show divergent DNA methylation patterns: Mammalian DNA methylation occurs mostly in the context of CG dinucleotides, whereas plant DNA methylation is prevalent in both CG and non-CG contexts, with the latter further classified into CHG (H = A, T, or C) and CHH DNA methylation (1). To date, how various DNA sequences interplay with DNA methyltransferases and other chromatin players to attain the dynamic DNA methylation landscape across the genome remains a long-standing question.

In plants, the establishment of DNA methylation in all sequence contexts is mediated by DOMAINS REARRANGED METHYLTRANSFERASE 2 (DRM2), following an RNA-directed DNA methylation pathway (4). Subsequently, CG methylation is maintained by plant DNA METHYLTRANSFERASE 1 (MET1) protein, the plant counterpart of DNMT1, CHG methylation is maintained by CHROMOMETHYLASE 3 (CMT3), and CHH methylation in long heterochromatic transposable elements (TEs) and short euchromatic TEs is maintained by CHROMOMETHYLASE 2 (CMT2) and DRM2, respectively (5, 6, 7, 8). Increasing evidence indicates that plant non-CG methylation is involved in transcriptional regulation and genome stabilization (9, 10). However, the mechanism by which non-CG methylation is established and maintained remains far from being clear.

Recent studies have demonstrated that non-CG methylation in plants exhibits a strong context bias (11, 12). Over three possible trinucleotide sequence motifs (CAG, CTG, and CCG) of CHG methylation, CWG (W = A or T) methylation is far more enriched than the CCG methylation in Arabidopsis, tomato, maize, and rice (12). Over nine possible trinucleotide sequence motifs (CAT, CTA, CAA, CTT, CCA, CCT, CAC, CTC, and CCC) of CHH methylation, CAA/CTA/CAT methylation shows a higher abundance than the rest in the same plants (12). Genome-wide mapping analysis indicated that the enriched CAA/CTA/CAT methylation is associated with both class I and II transposons, implicating their roles in transposon silencing (12). Our recent structural and biochemical characterization of CMT3 reveals a DNA intercalation interaction at the +1-flanking site, which energetically penalizes the rigid G/C nucleotides over flexible A/T nucleotides at the +1 position, resulting in an intrinsic enzymatic preference of CMT3 toward CWG motifs (13). Furthermore, it has been shown that the SRA domain of histone H3K9 methyltransferase KYPTONATE preferably binds to methylated CWG DNA (14). Such intrinsic enzymatic preference of the CHG methylation writer (CMT3) and binding specificity of the reader (KYPTONATE) together provide an explanation for the relative enrichment of the CWG methylation over CCG methylation in plants.

Likewise, our recent structural study of DRM2 methyltransferase (MTase) domain in complex with a subset of CHH (CTT and CAT) and CHG (CCG and CTG) substrates demonstrated that the DRM2-DNA interaction is also dominated by DNA intercalation: an arginine finger (R592) penetrates into DNA minor groove, prying open the base step between the guanine that normally pairs with the target cytosine in intact DNA and the +1-flanking nucleotide on the non-target strand (15). Such a R592-mediated DNA intercalation leads to a large conformational distortion at the CHH/CHG sites, creating a thermodynamic penalty for the rigid G/C nucleotides over flexible A/T nucleotide at the +1 position in a manner similar to that for CMT3 (15). In addition, the DNA shape beyond the CHH/CHG site contributes to the differential protein-DNA contacts among various DRM2-DNA complexes (15). On the other hand, due to the broad spectrum of CHH methylation, how the combined thermodynamic behaviors of the nucleotides at the +1- and +2-sites regulate DRM2-mediated DNA methylation across diverse CHH substrates remains unclear.

To gain a comprehensive understanding of the flanking sequence preference of DRM2, we set out to analyze the DNA methylation activity of DRM2 on CHH substrates under various sequence contexts. Our study revealed a combined effect of +1 and +2-flanking nucleotides on DRM2 activity, providing an explanation to the differential abundances of various CHH methylation in plants. Furthermore, we determined the crystal structure of the DRM2 MTase domain in complex with a CTA-containing DNA duplex, in which the target cytosine is followed by a highly flexible TpA step (16). Unlike what was observed for other DRM2-DNA complexes (15), introducing the TpA step next to the target cytosine results in two alternative conformations of the DRM2-bound DNA, which engage in different inter-base pair stacking as well as protein interactions. In line with the structural observation, mutational analysis of the DRM2 reveals a differential effect on DRM2-mediated CTA vs CTT methylation, supporting DNA conformational dynamics as another important factor in modulating DRM2-mediated DNA methylation. Furthermore, DRM2, but not the CTA DNA conformation-sensitive mutant, shows a temperature-dependent substrate preference for CTA over CTT DNAs. Together, our study demonstrates a thermodynamic view on how DNA deformation and base dynamics influence the DRM2-mediated CHH methylation, providing important mechanistic implications for the establishment and maintenance of context-biased DNA methylation in plants.

## Results

### DRM2-mediated DNA methylation over diverse CHH substrates

**Figure 1:**
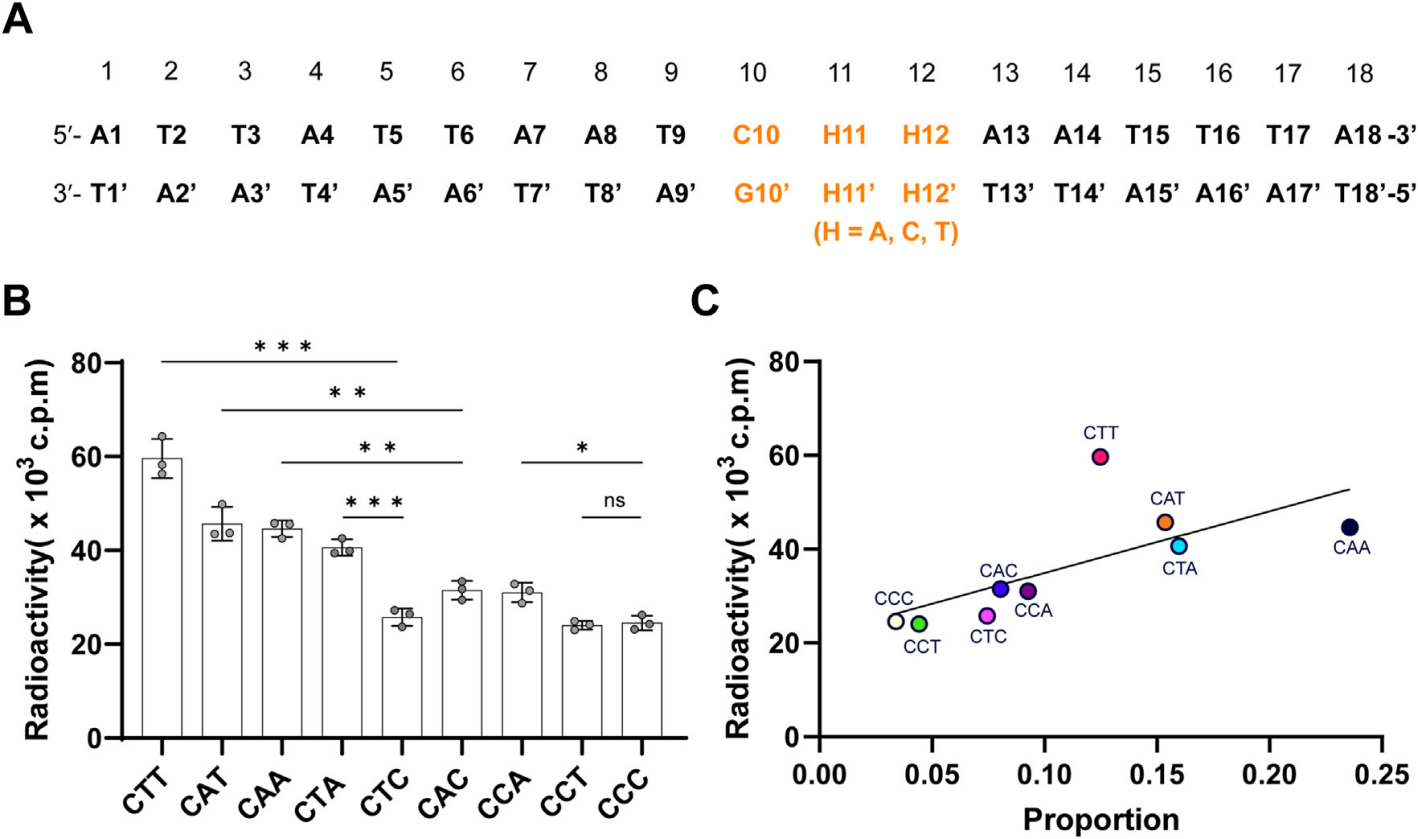
***In vitro* DNA methylation analysis of the DRM2-mediated CHH methylation.***A*, sequence for the 18-mer DNA duplexes used for DNA methylation assays. The CHH sites are colored in orange. *B*, *in vitro* DNA methylation assay of the DRM2 MTase domain on the CHH DNAs at 37 °C. Data are mean ± s.d. (n = 3 technical replicates). Statistical analysis for various CHH DNAs used a two-tailed Student’s *t* test. ns, not significant. ∗*p* < 0.05. ∗∗*p* < 0.01. ∗∗∗*p* < 0.001. *C*, correlation between DRM2-mediated CHH methylation *in vitro* and the relative abundance of mCHH motifs in differentially methylated cytosines identified from DRM2-complemented *drm1drm2cmt3 (ddc)*.

To gain insight into the relative activity of DRM2 over diverse CHH substrates, we performed *in vitro* DNA methylation assays of the DRM2 MTase domain on an 18-mer DNA duplex containing a varied central CHH motif at 37 °C (Fig. 1*A*). Under the experimental condition, there is a large variation of methylation efficiency of DRM2 MTase over the CHH substrates, with the methylation efficiency for the most favored DNA (CTT) greater than that for the least favored substrates (CCC, CTC and CCT) by nearly 3-fold (Fig. 1*B*). Further analysis of these methylation events revealed that these substrates largely fall into two groups with distinct methylation efficiency: the CTT, CTA, CAA, and CAT DNAs account for the group with high methylation efficiency, whereas the rest of the substrates, which contains at least one cytosine nucleotide at +1- and/or +2-flanking sites, accounts for the group with low methylation efficiency (Fig. 1*B*). This result suggests that the presence of A/T nucleotides at both the +1 and +2 sites, which creates a CWW motif, makes DNA a better substrate for DRM2. It is worth mentioning that the DNA methylation efficiency of DRM2 varies even within the group of CWW motifs. For instance, the methylation efficiency for CTT DNA appears more efficient than that for CAT, CTA, or CAA DNA. Together, these observations highlight a coordinated effect of the +1- and +2-flanking nucleotides on the methylation activity of DRM2.

To compare our *in vitro* observation with the relative enrichment of CHH DNA methylation in plants, we analyzed the CHH methylation efficiencies with differentially methylated cytosines (DMCs) identified in DRM2-complemented *drm1/drm2/cmt3* (*ddc*) *Arabidopsis* plants (15). We observed a strong correlation between the *in vitro* methylation efficiency of the CHH motifs and their relative abundance in DMCs: the CAA, CTA, CAT and CTT motifs that show high methylation efficiency *in vitro* account for the most frequent DMC events in plants, whereas the five CHH motifs with at least one cytosine nucleotide at +1 and/or +2 sites are clustered into a group with less frequent methylation events *in vitro* and *in vivo* (Fig. 1*C*). It is worth noting that the CTT motif, which is the most favored substrate of DRM2 *in vitro*, does not constitute the most abundant methylation sites in plants (Fig. 1*C*). Nevertheless, the strong correlation between *in vitro* methylation efficiency of the CHH motifs and their relative abundance in DMCs in plants suggests that the intrinsic methylation activity of DRM2 toward various CHH substrates may in part contribute to the context-dependent CHH methylation in plants.

### Structural overview of the DRM2-CTA DNA complex

**Figure 2:**
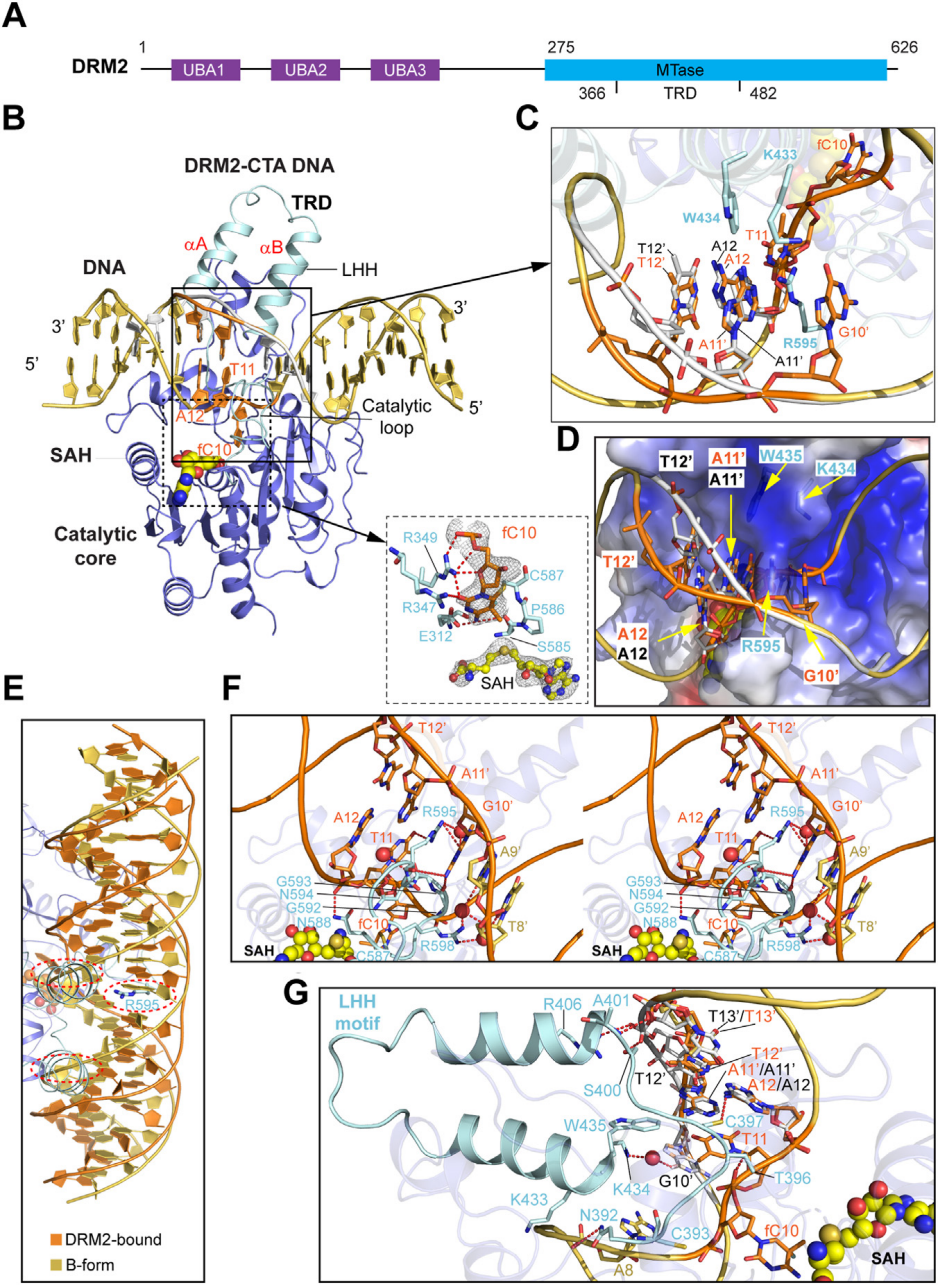
**Structural characterization of DRM2-CTA DNA complex.***A*, domain architecture of DRM2, with individual domains color-coded. The fragment used for crystallization is marked by *arrows* and labeled by residue numbers. TRD, target recognition domain (residues 366–482). *B*, ribbon representation of DRM2 MTase domain bound to the 18-mer CTA DNA duplex. The two subdomains of DRM2 MTase domain, catalytic core and TRD, are colored in pale *cyan* for the catalytic loop (residues 587–599) and the LHH motif, and in slate for the rest. The conformation I of the DNA is colored in yellow orange except for the (fC)TA site, which is colored in *orange*. The conformation II of the DNA is colored in *grey*. The two DNA-interacting helices in the LHH motif are labeled αA and αB, respectively. The SAH molecule is shown in sphere representation. The flipped-out fC10 and its interacting protein residues are shown in the expanded view, with the hydrogen bonds depicted as dashed lines. The Fo-Fc omit map for fC10 and the SAH molecule at 2σ contour level is shown as grey mesh. *C*, Close-up view of the two alternative conformations of the CTA site. The bulky DNA-interacting residues, K433, W434, and R595, are shown in stick representation. *D*, select region of the electrostatic surface of DRM2 bound to the CTA DNA, with interacting DRM2 residues and DNA nucleotides labeled. *E*, structural overlay of DRM2-bound CTA DNA and the structural model of B-form DNA in identical sequence, except that fC10 was replaced by a cytosine in the B-form DNA. The steric clashes between DRM2 regions (R595 and LHH motif) and the B-form DNA are indicated by dotted circles. *F*, stereo view of the interaction between the catalytic loop of DRM2 and DNA minor groove, with interacting residues and nucleotides labeled. *G*, close-up view of the interaction between the LHH motif of DRM2 and DNA major groove, with interacting residues and nucleotides labeled.

A/T-rich DNA sequence, which is associated with reduced helical stability and a strong tendency for deformation (17, 18, 19), has been recurrently involved in sequence-nonspecific protein interactions (16, 20). Furthermore, it has been established that the conformational dynamics of DNA is modulated by its sequence, with the TpA step as one of most flexible dinucleotide steps (16, 21, 22, 23, 24, 25, 26). In this regard, we asked how the DNA deformability of the flanking nucleotides interplays with base dynamics in controlling DRM2-mediated DNA methylation. To address this, we solved the crystal structure of the DRM2 MTase domain in complex with a DNA duplex mimicking the CTA DNA used for the *in vitro* DNA methylation assay (Fig. 1*A*), except that the target cytosine was replaced by a 5-fluorocytosine (fC) to ensure the formation of a stable, covalent complex between DRM2 and DNA (15). The crystal structure of the DRM2-CTA complex bound to cofactor by-product S-adenosyl-homocysteine (SAH) was refined to 2.91 Å resolution, with each asymmetric unit containing one complex (Fig. 2, *B* and *C* and Table 1).

**Table 1:** Crystallographic data collection and refinement statistics of the DRM2–CTA DNA complex

DNA complex	
Data collection	
Space group	*C 2 2 2*_*1*_
Cell dimensions	
*a*, *b*, *c* (Å)	54.5, 232.8, 118.5
α, β, γ (°)	90, 90, 90
Wavelength	0.9774
Resolution (Å)	48.41–2.91 (3.02–2.91)^a^
*R*_merge_	0.26 (0.82)
*I*/σ*I*	4.2 (1.13)
CC_1/2_	0.919 (0.487)
Completeness (%)	95.00 (88.11)
Redundancy	3.0 (2.6)
Total reflections	48,466 (3905)
Unique reflections	16,194 (1471)
Refinement	
No. reflections	16,153 (1467)
*R*_work_/*R*_free_ (%)	20.6/25.3
No. atoms	
Protein/DNA	3647
Ligand	104
Water	88
*B* factors (Å^2^)	
Protein	28.9
DNA	46.8
Ligand	25.7
Water	22.5
r.m.s. deviations	
Bond lengths (Å)	0.004
Bond angles (°)	0.73
Ramachandran	
Favored (%)	97.15
Allowed (%)	2.85
Outliers (%)	0.00

a Values in parentheses are for highest-resolution shell. The dataset was collected from a single crystal.

We were able to trace the entire MTase domain of DRM2 (residues 275–626) and the DNA molecule (Fig. 2*B*). As previously observed (15), The DRM2 MTase domain is composed of a catalytic core adopting a Rossmann fold and a loop-rich target recognition domain (TRD), creating a catalytic cleft to embed the DNA duplex (Fig. 2*B*). The target 5-fluorocytosine, fC10, flips into the catalytic pocket of DRM2, forming a covalent linkage with the catalytic cysteine C587 and hydrogen-bonding interactions with other catalytic residues (expanded view in Fig. 2*B*). Interestingly, the region encompassing Thy13′-Ade11′ on the non-target strand adopts two alternative conformations (Figs. 2*B* and S1, *A*–*E*), involving different helical configurations of the T12′pA11′ step (Fig. S1*C* vs *E*). As a result, the major conformation (conformation I, relative population of ∼0.55) manifests greater inter-strand distances for the T12′pA11′ step than the minor conformation (conformation II, relative population of ∼0.45) (Fig. S1, *B* and *D*).

The interaction between DRM2 and the CTA DNA is mediated by both the catalytic core and the TRD (Figs. 2, *B*–*G* and S2, *A* and *B*). Consistent with what was observed for other DRM2-DNA complexes (15), the catalytic loop (residues 585–596) of DRM2 enters the DNA minor groove, with residue R595 intercalating into the A11′pG10′ step on the non-target strand (Fig. 2, *C* and *D*) (15); on the other hand, a loop–helix (αA)–helix (αB) (LHH) motif of the TRD occupies the DNA major groove centered around the (fC)TA motif (Fig. 2*B*). Notably, residues K434 and W435 from the LHH motif approach the side chain of the DNA-intercalating R595 from the opposite direction, resulting in encirclement of the (fC)TA site (Fig. 2, *C* and *D*). Structural overlay of DRM2-bound CTA DNA with the structural model of the B-form CTA DNA reveals potential clashes between DRM2 R595 and the LHH motif and the non-distorted B-form DNA, explaining why formation of the productive DRM2-CTA DNA complexes led to DNA deformation (Fig. 2*E*).

### Structural details of the DRM2-DNA interaction

Similar to what was observed for other DRM2-DNA complexes (*e.g.* DRM2-CTT) (Fig. S2, *C* and *D*) (15), the catalytic loop of DRM2 mainly interacts with the (fC)TA motif on the target strand (Fig. 2*F*). Aside from the C587-fC10 covalent linkage, DRM2 R595 engages in base-specific interactions with Thy11 *via* a sidechain hydrogen bond and a water-mediated main-chain hydrogen bond, and the sidechain amino group of DRM2 N588 forms a hydrogen bond with the backbone phosphate of Ade12 (Fig. 2*F*). In addition, the catalytic loop makes contacts with Gua10′-Thy8′ on the non-target strand, with DRM2 G592 and R595 engaging in base-specific interactions with orphan Gua10′ *via* direct and water-mediated hydrogen bonds, respectively, and DRM2 R598 forming water-mediated hydrogen bonds with the −1/-2 flanking nucleotides (Ade9′ and Thy8′) on the non-target strand (Fig. 2*F*).

Toward the major groove, the TRD of DRM2 interacts with the (fC)TA site *via* the LHH motif: the sidechain amino group of DRM2 K433 forms a water-mediated hydrogen bond with the O6 atom of Gua10′, the indole ring of DRM2 W435 engages in van der Waals contacts with the base rings of Thy11 and Gua10′, the sulfhydryl group of DRM2 C397 is positioned within a hydrogen-bond distance with the N6 group of Ade12, and the side chain of DRM2 C393 engages in van der Waals contacts with the backbone of fC10. In addition, DRM2 S400, A401, R406 and K433 interact with the phosphate backbone of the DNA beyond the (fC)TA site *via* hydrogen-bonding and/or electrostatic interactions (Fig. 2*G*).

**Figure 3:**
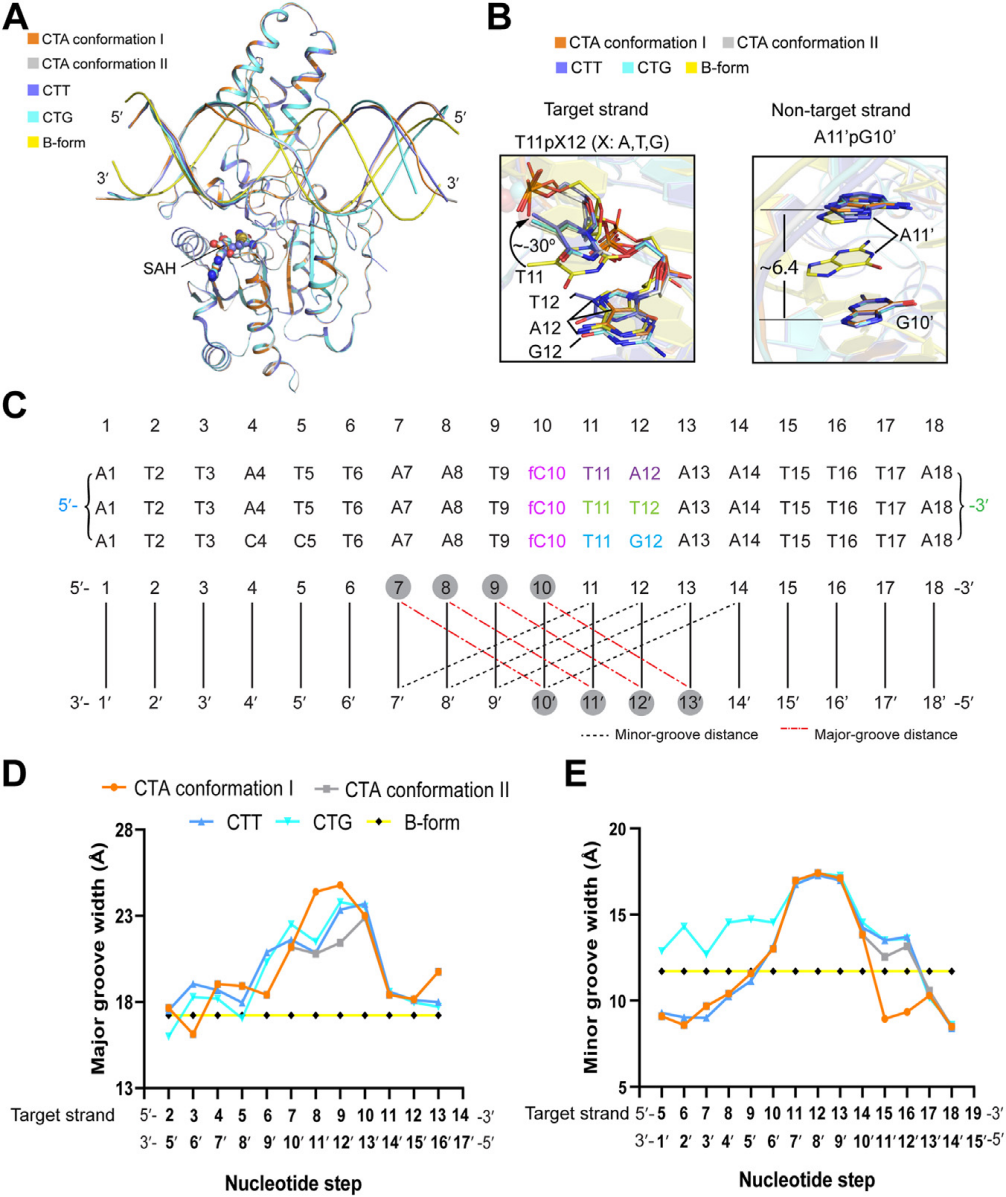
**Structural comparison of DRM2-CTA, DRM2-CTT and DRM2-CTG DNA complexes**. *A*, structural overlay of DRM2-CTA (Conformation I: *orange*; Conformation II: *grey*), DRM2-CTT (*slate*), and DRM2-CTG (*cyan*) DNA complexes. Also aligned is the B-form DNA (*yellow*) that possesses an identical sequence with the 18-mer CTA DNA. The SAH molecules are shown in sphere representation. *B*, (*left*) close-up view of the aligned +1- and +2-flanking sites on the target strand between the DRM2-CTA, DRM2-CTT, DRM2-CTG DNA complexes and the B-form DNA. (*right*) Close-up view of the aligned orphan guanine and +1-flanking site on the non-target strand between the DRM2-CTA, DRM2-CTT, DRM2-CTG DNA complexes and the B-form DNA. The inter-base distance for the A11′pG10′ step is indicated in unit of Å. *C*, DNA sequences for the CTA, CTT and CTG DNA bound to the DRM2 MTase domain. *D* and *E*, sequence-dependent major groove width (*D*) and minor groove width (*E*) measured for the DNA molecules bound to the DRM2 MTase domain.

It is worth noting that the DRM2-CTA DNA complex was crystalized under the same condition as previously reported DRM2-DNA complexes, resulting in nearly identical space group parameters (15) (Table 1). Nevertheless, our comparative structural analysis revealed that only the conformation II of the DRM2-CTA DNA complex resembles the previously determined DRM2-DNA complexes (Fig. 3*A*) (27), whereas the conformation I is uniquely observed in the DRM2-CTA DNA complex. This observation highlights an effect of DNA conformational dynamics on the DRM2-DNA interactions.

### DRM2-DNA complexes exhibit a conserved DNA deformation mechanism

To illustrate how the +1- and +2-flanking nucleotides collectively influence DRM2-mediated DNA methylation, we superimpose the structure of the DRM2-CTA DNA complex with those of DRM2-CTT and DRM2-CTG DNA complexes as determined previously (15) (Fig. 3, *A* and *B*). Note that the CTA DNA differs from the CTT DNA only at the +2-flanking site, in which the A12⋅T12′ pair in the CTA DNA is replaced by the T12⋅A12′ pair in the CTT DNA, and both CTT and CTA DNAs differ from the CTG DNA in the −6 and −5 flanking sites, in which A4⋅T4′ and T5⋅A5′ pairs in CTT/CTA DNAs are replaced by C4⋅G4′ and C5⋅G5′ pairs, respectively, in CTG DNA (Fig. 3*C*). Nevertheless, DRM2-CTA is well aligned with both DRM2-CTT and DRM2-CTG complexes, with a root-mean-square deviation (RMSD) of 0.15 Å and 0.18 Å over 310 and 308 aligned Cα atoms, respectively (Fig. 3*A*), consistent with the fact that these complexes share a similar pattern of protein-DNA interactions.

Further comparison of the DRM2-CTA, DRM2-CTT, and DRM2-CTG DNA complexes revealed that the +1- and +2-flanking sites (T11pX12 step, X=A, T, G) on the target strand undergoes similar DNA deformation (Fig. 3*B*). Common to all the DRM2-DNA complexes (Fig. 3*B*), DRM2 R595-mediated DNA intercalation increased the rise between the A11′pG10′ step by ∼6.4 Å and introduced a roll to Thy11 by around −30° (Figs. 3*B* and S1, *C* and *E*), resulting in enlargement of major groove and minor groove in a similar fashion among the three complexes (Fig. 3, *C*–*E*). As noted previously (15), an earlier study indicated that the four CpX (X denotes A, T, C or G) dinucleotides are mediated by a different extent of inter-base pair stacking interaction, with the CpG and CpC steps associated with relatively high stacking energy (28). Along this line, the DRM2 binding-induced DNA deformation, which reduces the inter-base pair interactions at the CHH sites, provides a mechanism in discriminating flexible A/T nucleotides over rigid G/C nucleotides (29). The fact that the DRM2-DNA complexes share a similar DNA deformation mechanism reinforces the notion that DNA deformation contributes to the substrate preference of DRM2 toward A/T nucleotides at the +1 and +2 positions. A detailed thermodynamic analysis of the DRM2-CHH DNA contacts awaits further investigation.

As noted above, the major difference between the DRM2-CTA, the DRM2-CTT and the DRM2-CTG complexes lies in the region corresponding to the CHH site: unlike the DRM2-CTT and DRM2-CTG complexes each dominated by one conformation, the DRM2-CTA complex adopts two alternative conformations in this region (Fig. 3, *A* and *E*). In comparison with the conformation II that aligns well with the corresponding region of CTT and CTG complex, the conformation I compresses the T13′pT12′ step further toward the minor groove, resulting in a reduced minor groove width of this region (Fig. 3*E*). Beyond the central CHH sites, the DRM2-bound CTG DNA shows an enlarged minor groove width for T5-T9 over the corresponding region in the DRM2-bound CTT and CTA DNA (Fig. 3*E*), in line with the sequence variation of CTG DNA from the CTT and CTA DNA (Fig. 3*C*). This observation reinforces a previous notion that DNA shape plays a role in protein-DNA interaction and that G/C nucleotides tend to create a wider minor groove than T/A nucleotides (29).

### Effect of DNA conformational dynamics on the DRM2-DNA interaction

**Figure 4:**
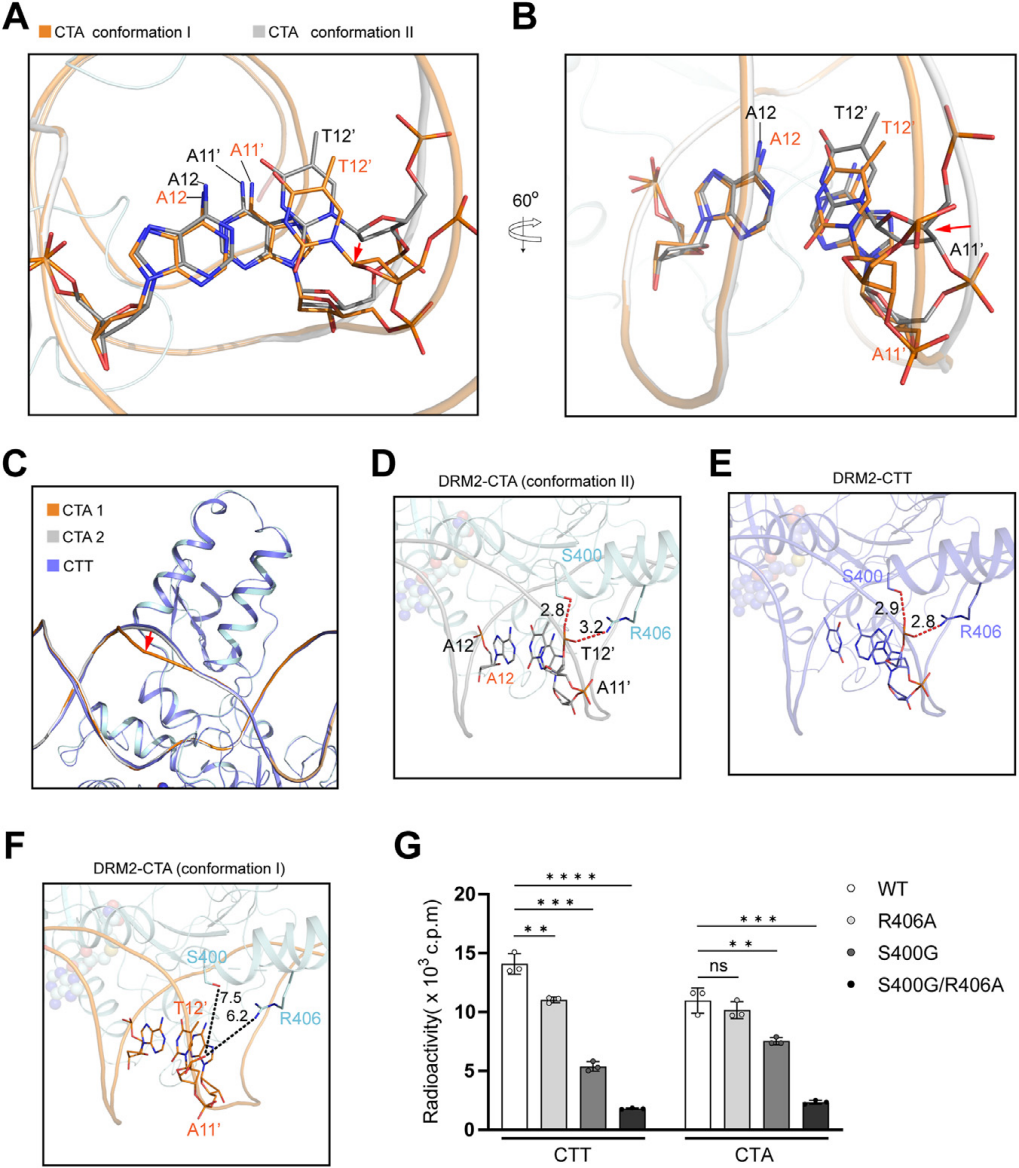
**Structural and biochemical analysis of the two alternative DNA conformations of the DRM2-CTA complex.***A* and *B*, two different views of the DNA conformations at the +1- and +2-flanking sites. *C*, close-up view of the interaction between the DRM2 LHH motif and DNA in the DRM2-CTA and DRM2-CTT DNA complexes. The positioning of DNA backbone in the conformation II of the DRM2-CTA complex relative to that of the conformation I is indicated by *red arrow*. *D*–*F*, Close-up view of the interaction between DRM2 S400/R406 and DNA in the conformation II of the CTA complex (*D*), the CTT complex (*E*), and the conformation I of the CTA complex (*F*). The distances between the interaction sites are indicated in unit of Å. *G*, *In vitro* DNA methylation assays of WT, S400G-, R406A- or S400G/R406A-mutated DRM2 on CTA or CTT DNA at 37 °C. Statistical analysis for WT vs mutants used two-tailed Student’s *t* test. Data are mean ± s.d. (n = 3 technical replicates). ns, not significant. ∗*p* < 0.05; ∗∗∗*p* < 0.001.

Detailed comparison of the two alternative conformations of the DRM2-bound CTA DNA reveals that Thy12′ undergoes a negative propeller twist from conformation II to conformation I (36.5° vs 29.6°) (Figs. 4, *A* and *B* and S1, *C* and *E*), resulting in a greater inter-base pair stacking for the T12′pA11′ step (136 Å^2^ in conformation I vs 121 Å^2^ in conformation II) (Fig. 4, *A* and *B*). Such a difference in base-stacking interaction between the two conformational states is accompanied by a differential interaction between the CTA DNA and the TRD of DRM2: In conformation II, Thy12′ and Ade11′ are in proximity with the TRD, reminiscent of what was observed for the DRM2-CTT complex (Fig. 4*C*). Accordingly, the side chains of DRM2 S400 and R406 engaged in hydrogen-bonding interactions with the backbone phosphate of Thy12′ in the conformation II (Fig. 4*D*), as observed for that in the DRM2-CTT complex (Fig. 4*E*). In contrast, the corresponding region in the conformation I moves away from TRD by ∼4 Å, resulting in an increase of the distance between S400/R406 and the backbone of Thy12′ to 6.2 Å and 7.5 Å, respectively, in the conformation I (Fig. 4, *C* and *F*). These conformational changes collectively lead to a reduced protein-DNA interaction interface (buried surface area of 1664 Å^2^) for the conformation I than that for the conformation II (buried surface area of 1727 Å^2^) (Fig. 4*C*).

The observation that the interaction between DRM2 S400/R406 and DNA is present in the CTT DNA complex and the conformation II of the CTA DNA complex, but not in conformation I of the CTA DNA complex, raises a possibility that this interaction may impact differentially on DRM2-mediated methylation of the CTT *versus* CTA DNA. To test this possibility, we performed *in vitro* DNA methylation assay of DRM2, WT, S400G-, R406A-,or S400G/R406A-mutated, on CTT and CTA DNAs at 37 °C. Wild-type (WT) DRM2 shows significantly higher methylation efficiency than the S400G or R406A mutant on the CTT DNA (Fig. 4*G*), consistent with the fact that DRM2 S400 and R406 are involved in the interaction with the CTT DNA (29) (Fig. 4*E*). In contrast, WT, S400G and R406A DRM2 show a much-reduced difference in their methylation efficiency on the CTA DNA (Fig. 4*G*). This observation suggests that the S400G or R406A mutation affects the DRM2-mediated DNA methylation on the CTT substrate to a greater extent than it on the CTA substrate, in line with the fact that the DRM2 S400- and R406-mediated DNA interaction is stable in the CTT DNA complex, but partly disrupted in the conformation II of the CTA DNA complex (Fig. 4, *D*–*F*). It is worth noting that the S400G/R406A double mutation led to a greater activity reduction on both DNA substrates than either S400G or R406A mutation; nevertheless, like the S400G and R406A mutations, the S400G/R406A double mutation appears to impair the CTT methylation more than the CTA methylation (Fig. 4*G*). Together, these data support the notion that the conformational dynamics of DNA influences DRM2-mediated DNA methylation.

### DRM2 shows a temperature-dependent substrate preference for CTA over CTT DNA

**Figure 5:**
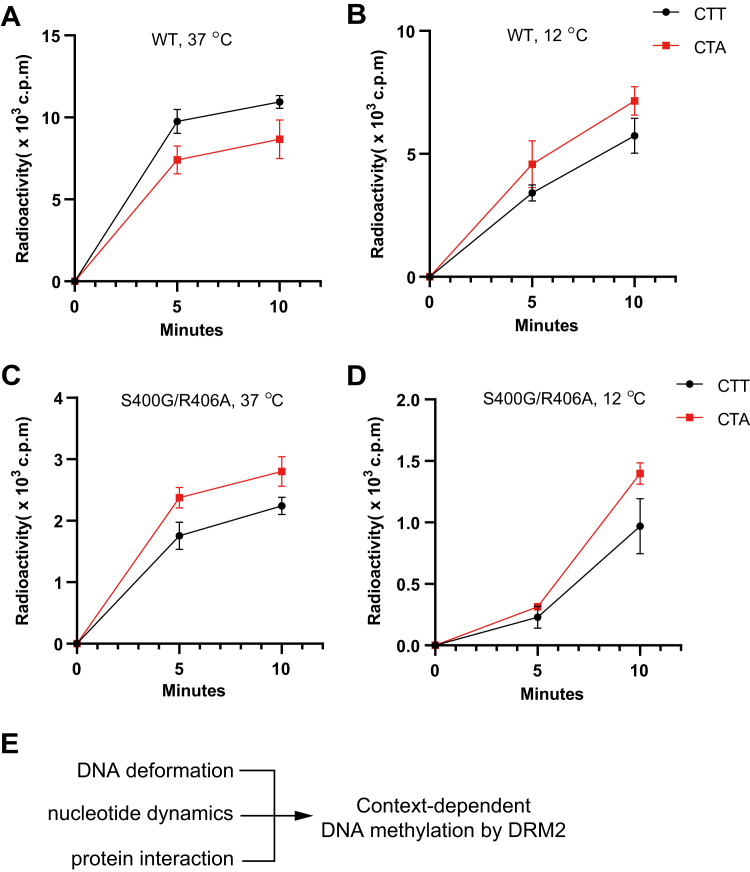
**DNA conformational dynamics interplay with temperature in regulating the substrate preference of DRM2.***A* and *B, in vitro* DNA methylation kinetics of WT DRM2 MTase domain on CTT and CTA DNAs at 37 °C (*A*) and 12 °C (*B*). *C* and *D*, *in vitro* DNA methylation kinetics of S400G/R406A DRM2 MTase domain on CTT and CTA DNAs at 37 °C (*C*) and 12 °C (*D*). Data are mean ± s.d. (n = 3 technical replicates). *E*, a working model for the thermodynamic factors that contribute to the context-dependent DNA methylation by DRM2.

The observation that the CTA and CTT DNAs give rise to a different extent of conformational dynamics in their respective DRM2-bound states raises a question on whether DRM2-mediated CTA and CTT DNA methylation involve different activation energies. To explore this, we measured the *in vitro* DNA methylation kinetics of DRM2 on CTA and CTT DNAs at two different temperatures, 12 °C and 37 °C. Consistent with the observation above (Fig. 4*G*), WT DRM2 methylated the CTT DNA more efficiently than the CTA DNA at 37 °C (Fig. 5*A*). However, lowering temperature from 37 °C to 12 °C led to switch of the preferred substrate of DRM2 from CTT to CTA DNA (Fig. 5*B*), suggesting that decrease in the conformational dynamics of the CTA DNA at 12 °C might lower the corresponding entropic penalty for the enzyme-substrate association. In contrast, S400G/R406A-mutated DRM2 shows a consistent preference for CTA over CTT DNA at both temperatures (Fig. 5, *C* and *D*), in line with the observation that the S400G/R406A mutation mainly impairs the CTT DNA complex and the conformation II of the CTA DNA complex, but not the conformation I of the CTA DNA complex. In this regard, our thermal shift analysis revealed a nearly identical melting temperature between WT DRM2 and the S400G/R406A mutant (Fig. S3, *A* and *B*), thereby ruling out the possibility that their different enzymatic behavior is attributed to a change of protein stability. Together, these data establish a link between the conformational dynamics of DNA substrates and temperature-dependent substrate preference of DRM2, which may contribute to the temperature-dependent shift of non-CG DNA methylation landscape that occurs in plants (30, 31).

## Discussion

Prevalent in plant genomes, non-CG DNA methylation constitutes an epigenetic landscape that is highly sequence context-dependent and important for TE silencing and genome stability. DRM2 has been established as a critical player in the maintenance of CHH methylation in plants. DRM2-mediated DNA methylation is subject to regulation by small RNAs, histone modifications, non-coding RNA, and protein interactions (32, 33, 34, 35). To date, how the intrinsic enzymatic property of DRM2 interplays with various chromatin factors to orchestrate specific DNA methylation patterns across the genome remains unclear. This study, through enzymatic analysis of DRM2 over diverse CHH motifs, structure determination of DRM2 with a CTA DNA substrate, and mutational analysis, reveals how the conformational dynamics of DNA substrate influences DRM2-mediated DNA methylation, with important implications in plant DNA methylation establishment and maintenance and its interplay with environment.

First, this study unravels a link between the intrinsic DNA methylation activity of DRM2 over the broad spectrum of DRM2-mediated CHH methylation in plants. Through comparative enzymatic analysis of the CHH substrates, this study reveals a marked substrate preference of DRM2 toward CWW motifs, suggesting that the intrinsic DNA methylation specificity of DRM2 may contribute to the context bias of CHH methylation *in vivo*. Furthermore, this study shows that DNA deformation represents a conserved feature of the DRM2-CHH DNA complexes. The DNA intercalation interaction by residue R595 leads to the disruption of the inter-base pair stacking between the guanine that would otherwise pair with the target cytosine and the +1-flanking nucleotide on the non-target strand, as well as between the +1- and the +2-flanking nucleotides on the target strand. Observation of such a distorted DNA conformation was enabled by introducing an irreversible covalent linkage between DRM2 C587 and the fC10 nucleotide in this study. These DNA distortions conceivably create a conformational penalty that serves to discriminate nucleotide composition, thereby providing an explanation for the context-dependent CHH methylation by DRM2 (Fig. 5*E*). This DNA deformation-regulated DRM2 activity is reminiscent of what was observed for CMT3-mediated CHG methylation (13), and more broadly, the recurrent transcription factor-DNA interactions that involve DNA distortion (36).

DNA conformation has been well recognized as an important factor in modulating protein-DNA interaction (37). The DNA sequence-encoded conformational parameters, such as major groove and minor groove widths, profoundly influence the binding specificity and affinity of proteins (16, 29). Furthermore, a large body of computational, structural, and biochemical analyses have linked DNA sequence to its conformational dynamics, with the TpA step identified as one of the most flexible nucleotide steps (16, 21, 22, 23, 24, 25, 26). Along this line, this study reveals that the CTA-containing DNA adopts two alternative conformations when interacting with DRM2. In comparison with the conformation II, the conformation I involves a greater inter-base pair stacking at the +1/+2-flanking sites, but a reduced DRM2-DNA interaction. Our combined mutational and enzymatic analysis further demonstrates that this DNA conformational dynamics contributes to the context-dependent DNA methylation by DRM2 (Fig. 4*H*). Remarkably, the relative DNA methylation substrate preference of WT DRM2, but not the S400G/R406A mutant, for the CTT and CTA DNAs became switched when the reaction temperature was decreased from 37 °C to 12 °C, suggesting that the interplay between the conformational dynamics of DNA substrates and temperature as an environmental factor may contribute to the temperature-dependent variation of the non-CG DNA methylation that occurs in plants (30, 31). A more comprehensive understanding of how thermodynamic behaviors of a DRM2-DNA complex crosstalk with environmental factors in regulating plant DNA methylation awaits further investigation.

In summary, this study provides both structural and dynamic insights into the DRM2-mediated CHH methylation, highlighting the intricate interplay between DNA methylation machinery, DNA deformation, and base dynamics that give rise to a complex CHH methylation landscape in plants.

## Experimental procedures

### Protein expression and purification

The expression plasmid for the DRM2 MTase domain was generated as described before (15). In essence, a synthetic DNA fragment encoding the MTase domain of *Arabidopsis thaliana* DRM2 (residues 270–626) was cloned into a modified pRSFDuet-1 vector (Novagen), in which the DRM2 MTase DNA sequence was separated from the His6-SUMO tag by a ubiquitin-like protease 1 (ULP1) cleavage site. The expression plasmid was transformed into *E. coli* BL21 DE3 (RIL) cells, which were then cultured at 37 °C. After the cell density reached an optical density at 600 nm of 0.8, the temperature was lowered to 16 °C and the cells were induced by 1 mM isopropyl-D-thiogalactopyranoside and continued to grow overnight. The cells were collected, resuspended in lysis buffer [50 mM tris-HCl (pH 8.0), 1 M NaCl, 25 mM imidazole, and 1 mM phenylmethylsulfonyl fluoride], and lysed using an Avestin Emulsiflex C3 homogenizer. After centrifugation, the supernatant was loaded to a Nickel–NTA affinity column (GE Healthcare) and the His6-SUMO-DRM2 fusion protein was eluted with elution buffer [20 mM tris-HCl (pH 8.0), 350 mM NaCl, and 300 mM imidazole]. The His6-SUMO tag was then removed by ULP1–mediated cleavage. The tag-free protein was further purified through ion-exchange chromatography on a Heparin HP column (GE Healthcare) and size-exclusion chromatography on a 16/600 Superdex 200 pg column (GE Healthcare). The final protein sample was concentrated and stored in −80 °C freezer for future use.

### Assembly of the covalent DRM2-CTA complex

To generate covalent DRM2-CTA complex, DRM2 MTase reacted with a synthesized 18-mer DNA duplex (Keck Biotechnology Resource Laboratory, Yale University) containing a central CTA motif, in which the target cytosine is replaced by 5-fluorodeoxycytosine (5′-ATTATTAATXTTAATTTA-3′; X = 5-fluorodeoxycytosine), in a buffer containing 25 mM Tris-HCl (pH 8.0), 25% glycerol, 50 mM dithiothreitol (DTT), and 30 mM *S*-adenosyl-L-methionine (SAM) at room temperature. The reaction products were sequentially purified through ion-exchange chromatography on a HiTrap Q XL column (GE Healthcare) and size-exclusion chromatography on a 16/600 Superdex 200 pg column. The final protein sample was concentrated to ∼0.5 mM in a buffer containing 20 mM Tris-HCl (pH 8.0), 250 mM NaCl, 5 mM DTT, and 5% glycerol.

### Crystallization conditions and structure determination

For crystallization, 0.2 to 0.3 mM DRM2-CTA complex was mixed with 1 mM SAH. Crystals for the DRM2-DNA complex were generated using sitting-drop vapor-diffusion method at 4 °C. Each drop was prepared by mixing 0.5 μl of the DRM2-CTA complex sample with 0.5 μl of precipitant solution [0.2 M Ammonium citrate tribasic and 20% w/v polyethylene glycol 3350 (pH 7.0)]. The crystal quality was further improved using the microseeding method. To harvest crystals, the crystals were soaked in cryoprotectants made of mother liquor supplemented with 30% glycerol before flash-frozen in liquid nitrogen. The X-ray diffraction dataset for the DRM2-CTA complex was collected on beamline 5.0.1 at the Advanced Light Source, Lawrence Berkeley National Laboratory. The diffraction data were indexed, integrated, and scaled using the HKL-3000 program (38). A moderate overall I/σI (4.2) was obtained, owing to the relatively small crystal size. Nevertheless, a diffraction resolution of 2.91 Å was selected based on the CC_1/2_ value of 0.487 for the highest-resolution shell. The structure was solved by molecular replacement with the PHASER program (39) using the structure of DRM2-CTT complex (Protein Data Bank: 7L4C) as search model. The structural model of the DRM2-CTA complex was subjected to iterative modification using COOT (40) and refinement using the PHENIX software package (41). The same R-free test set was used throughout the refinement. The statistics for data collection and structural refinement of the covalent DRM2-CTA complex are summarized in Table 1.

### *In vitro* DNA methylation assay

*In vitro* methylation assay was performed in 20-μL reactions containing 1 μM DRM2 (wild type or mutant), 3 μM synthesized DNA duplexes, 0.56 μM S-adenosyl-L-[methyl-3H] methionine with a specific activity of 18 Ci/mmol (PerkinElmer), 1.96 μM nonradioactive SAM, 50 mM Tris-HCl (pH 8.0), 0.05% β-mercaptoethanol, 5% glycerol, and bovine serum albumin (BSA; 200 μg/ml). The CHH DNA substrates are made of 18-mer DNA duplex (target strand: 5′-ATTATTAATCHHAATTTA; H = A, T or C), harboring CTA, CTT, CTC, CAA, CAT, CAC, CCA, CCT or CCC motif. For the CCC, CCT, CCA, CAC and CTC substrates, the cytosine(s) aside from the target cytosine is replaced with a 5-methylcytosine. After annealing, formation and quality of the DNA duplex were confirmed on a 10% native acrylamide gel. Reactions were incubated at 37 °C or 12 °C for various durations: 20 min for activity comparison of DRM2 among all possible CHH motifs (Fig. 1*C*), 10 min for activity comparison of WT and mutant DRM2 over CTT and CTA DNAs (Fig. 4*G*), and varied durations (0, 5, 10 min) for the kinetic measurements (Fig. 5, *A*–*D*), before being quenched by the addition of 5 μl of 10 mM nonradioactive SAM. The reaction mixtures (10 μl) were then loaded onto a DEAE membrane (PerkinElmer) and air dried. The membrane was washed with 0.2 M ammonium bicarbonate (pH 8.2) two times for 15 min each, deionized water once for 15 min, and 95% ethanol once for 15 min. After air drying, the membrane was transferred into vials containing 4 ml of scintillation buffer (Fisher) and subjected to tritium scintillation recording by a Beckman LS6500 counter. Each reaction was replicated three times. For control, all the methylation assays included samples containing DRM2 and SAM only in the reaction buffer, which gave basal levels of radioactivity to be subtracted from the actual reaction readings for data analysis.

### Thermal shift assay

Thermal shift assays were performed for WT and S400G/R406A DRM2 proteins using a Bio-Rad CFX Connect Real-Time PCR Detection System. Each sample was made of 1 μM DRM2 protein dissolved in a buffer containing 50 mM Tris-HCl (pH 8.0), 0.05% β-mercaptoethanol, 5% glycerol, and 1X GloMelt Dye (Biotium). The plate containing samples in triplicate was gradually heated from 4 °C to 95 °C with an increment step of 0.5 °C. Fluorescence intensities were recorded within the excitation and emission wavelengths of 470 and 510 nm, respectively.

### Analysis of differentially methylated cytosines

A custom Perl script was used to locate the CHH motifs and print the +1- and +2-flanking sites on the Arabidopsis TAIR10 reference genome. Differentially methylated cytosines (DMCs) were called as previously described using the deposited bisulfite-sequencing data in NCBI Gene Expression Omnibus (accession number GSE146700) (15). In essence, DMCs were identified using both MethylKit (42) and bsmap’s methdiff.py script with the 10% cutoff, and only overlapped ones were used for analysis.

### Statistics

The two-tailed Student’s *t* tests were performed to compare distributions between different groups. The *p* value lower than 0.05 was considered to be statistically significant.

## Data availability

Coordinates and structure factors for the DRM2-CTA complex have been deposited in the Protein Data Bank under accession code 8T1U.

## Supporting information

This article contains supporting information.

## Conflict of interest

The authors declare that they have no known competing financial interests or personal relationships that could have appeared to influence the work reported in this paper.
