# Supplementary material for: DNA conformational dynamics in the context-dependent non-CG CHH methylation by plant methyltransferase DRM2
Source: J Biol Chem. 2023 Nov 4;299(12):105433. doi: 10.1016/j.jbc.2023.105433 (PMC10711165; doi:10.1016/j.jbc.2023.105433)
Supplement: Supporting information [file mmc1.pdf]

Supplementary information for

DNA conformational dynamics in the context-dependent non-CG CHH methylation by plant methyltransferase DRM2

Jianbin Chen[1], Jiuwei Lu[1], Jie Liu[2], Jian Fang[1], Xuehua Zhong[2], Jikui Song[1,#]

[1]Department of Biochemistry, University of California, Riverside, CA 92521, USA

[2]Biology Department, Washington University in St. Louis, St. Louis, MO 63130-4899

[#]Correspondence: jikui.song@ucr.edu

A

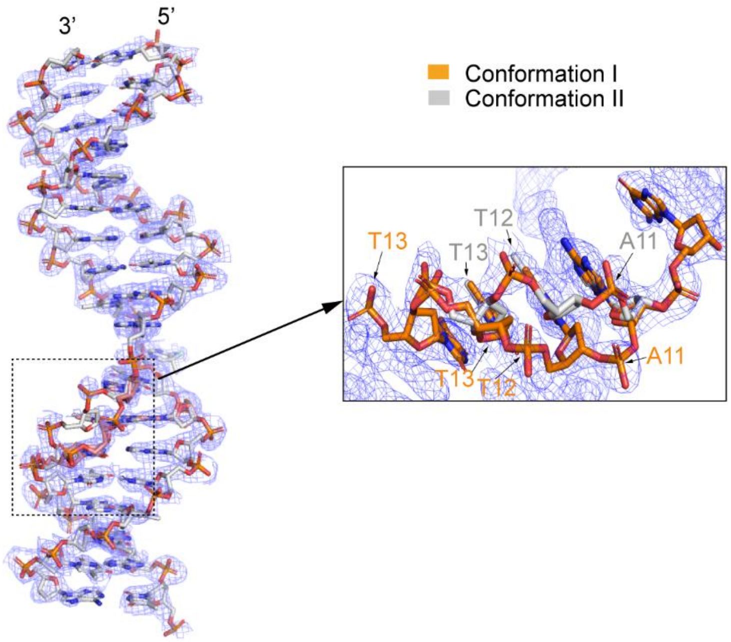

B

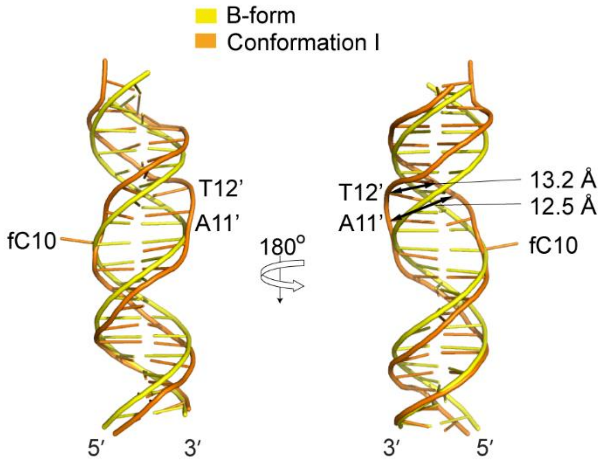

C

Step	Rise (Å)	Tilt (°)	Roll (°)	Twist (°)
A11′pG10′	6.4	6.2	11.1	-4.5
T12′pA11′	3.8	0.7	-0.3	29.6
T11pA12	3.3	6.5	-24.3	41.2
B-form	3.3	4.4	1.8	35.2

Pair	Buckle(°)	Propeller(°)	Opening(°)
T11-A11′	7.8	12.2	2.4
A12-T12′	-2.1	-10.7	13.5
B-form	0	-14.5	-1.1

D

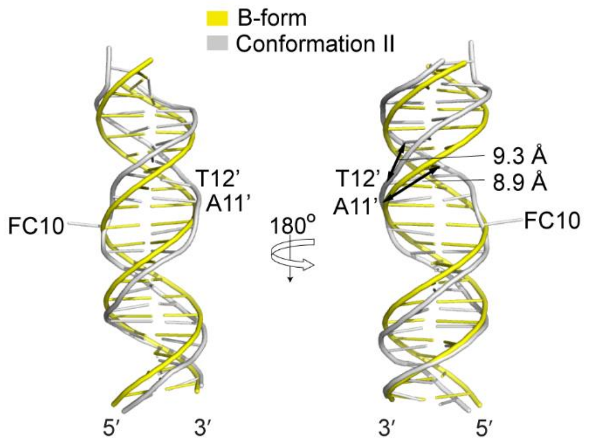

E

Step	Rise (Å)	Tilt (°)	Roll (°)	Twist (°)
A11′pG10′	6.5	2.7	12.8	0.8
T12′pA11′	3.6	3.8	-2.3	36.5
T11pA12	3.9	1.9	-30.3	44.6
B-form	3.3	4.4	1.8	35.2

Pair	Buckle(°)	Propeller(°)	Opening(°)
T11-A11′	10.1	11.1	-3.6
A12-T12′	4.6	-13.2	5.3
B-form	0	-14.5	-1.1

Figure S1. Biochemical and structural analysis of DRM2-mediated CHH DNA methylation. (A) Fo-Fc omit map for the DRM2-bound CTA is shown as blue mesh contoured at 2σ level. The conformational difference between the two alternative conformations of the DNA is highlighted in expanded view, with the corresponding nucleotides labeled. (B) Structural overlay between the CTA DNA in the conformation I and the B-form DNA in identical sequence (except that the fC10 is replaced by a cytosine in the B-form). The inter-strand distances for the T12'pA11' step are indicated. (C) (top) Base step parameters for the CTA site of the conformation I. (bottom) Base-pair parameters for the CTA site of the conformation I. (D) Structural overlay between the CTA DNA in the conformation II and the B-form DNA in identical sequence (except that the fC10 is replaced by a cytosine in the B-form). The inter-strand distances involving the T12'pA11' step are labeled. (E) (top) Base step parameters for the CTA site of the conformation II. (bottom) Base pair parameters for the CTA site of the conformation II.

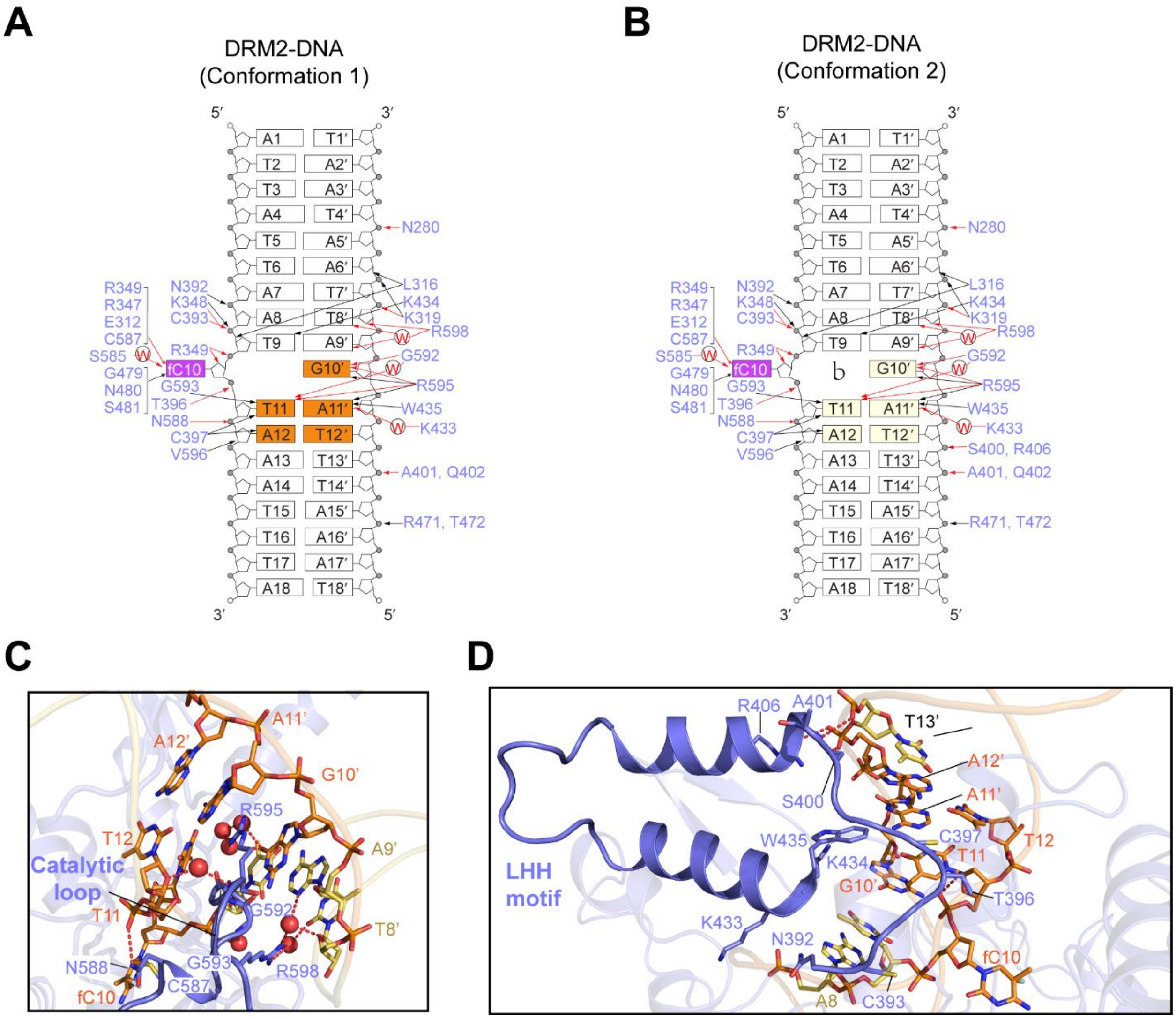

Figure S2. Structural analysis of the DRM2-CTA DNA interaction. (A,B) Schematic view of the interaction between DRM2 MTase and CTA DNA in conformation I (CTA1) (A) and conformation II (CTA2) (B). The hydrogen bonds and electrostatic contacts were indicated by red and van der Waals contacts are indicated by red and black arrows, respectively. Water-mediated hydrogen bonds are denoted by letter “W”. (C,D) For comparison with the DRM2-CTA DNA, close-up view of the DRM2-CTT DNA interactions in the catalytic loop (C) and the LHH motif of the TRD (D). Hydrogen bonds are depicted as dashed lines.

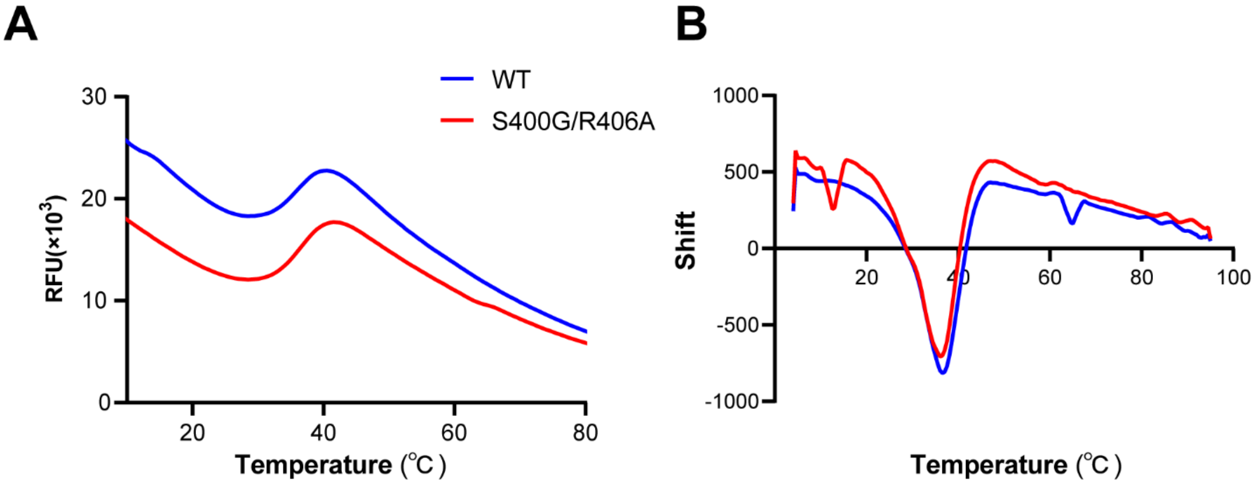

Figure S3. Thermal shift analysis of WT and mutant DRM2. (A,B) Thermal shift assay for the WT and S400G/R406A DRM2 MTase domain, with raw fluorescence data (A) and first derivative of the raw data (B) shown.
